# Conducting rigorous implementation evaluations in real word settings: lessons from a consensus approach to perioperative pathway implementation for elective surgery

**DOI:** 10.1186/s43058-026-00876-4

**Published:** 2026-02-06

**Authors:** Lisa Pagano, Andrew Hirschhorn, Gaston Arnolda, Janet C. Long, Emilie Francis-Auton, Jeffrey Braithwaite, Kate Churruca, Louise A. Ellis, Peter D. Hibbert, Andrew Partington, Marcus Stoodley, Mitchell N. Sarkies

**Affiliations:** 1 Australian Institute of Health Innovation, Faculty of Medicine, Health and Human Sciences, Macquarie University, Sydney, Australia; 2 Faculty of Medicine, Health and Human Sciences, MQ Health, Macquarie University, Sydney, Australia; 3 Impact in Health, Allied Health and Human Performance, University of South Australia, Adelaide, Australia; 4 Flinders Health and Medical Research Institute, Flinders University, Adelaide, Australia; 5 School of Health Sciences, Faculty of Medicine and Health, University of Sydney, Sydney, Australia; 6 Implementation Science Academy, Sydney Health Partners, University of Sydney, Sydney, Australia

**Keywords:** Implementation science, Consensus, Evaluation, Perioperative care, Health Services Research

## Abstract

**Introduction:**

Single site quasi-experimental implementation studies provide opportunities to learn about implementation in context. There is limited guidance on how to best utilise these studies to maximise opportunities for learning at scale. This study evaluated the use of a consensus process to develop and implement standardised perioperative pathways, and aimed to provide practical insights on conducting rigorous, theory-informed evaluations that can generate transferable insights for implementation science.

**Methods:**

A multi-method quasi-experimental study was conducted in a private hospital in Australia. Six consensus-based surgical care pathways were developed and implemented by different clinical teams, following a four-stage implementation process using the Exploration, Preparation, Implementation and Sustainment (EPIS) framework. Implementation outcomes were explored through participant observations (16 h) and semi-structured interviews (*n* = 9), which were analysed thematically using an interpretive descriptive approach. Normalisation Process Theory (NPT) was then applied to understand the mechanisms of change in greater depth. Pathway fidelity was assessed via medical record audits from a random patient sample (*n* = 90) from four surgical cohorts.

**Results:**

Implementing standardised perioperative pathways using a multi-faceted consensus-based implementation plan was perceived as acceptable, appropriate, and feasible. However, fidelity to clinical actions improved in only two of four surgical cohorts. Implementation was operationalised through the four generative mechanisms of NPT and was influenced by factors that related to all four constructs and 12/16 elements of the EPIS framework. Factors relating to the Inner Context and the Innovation were most frequently identified as having a greater influence on implementation across all EPIS phases. The implementation plan targeted Collective Action and Coherence to a greater extent than other mechanisms. Participants linked greater uptake and implementation to the importance of co-designing implementation strategies with frontline staff (improving Legitimation and Coherence) and tailoring strategies to specific disciplines.

**Conclusions:**

This project provides a practical case study for how to undertake theory-informed, implementation evaluations in real-world contexts. It offers valuable insights for others seeking to operationalise implementation science principles in everyday healthcare settings including how individual strategies may work to drive local change.

**Supplementary Information:**

The online version contains supplementary material available at 10.1186/s43058-026-00876-4.

Contributions to the literature

- We provide an example of how a theory-informed, multi-method evaluation embedded within a quasi-experimental study can generate valuable conceptual and empirical insights into how implementation unfolds in context.
- We demonstrate that small, single site, local quality improvement initiatives can yield generalisable insights when evaluated rigorously, using multi-methods and building on and integrating existing frameworks.
- By examining local consensus discussions, we illustrate how Normalisation Process Theory mechanisms operate within a single strategy, illuminating their role in shaping coherence, participation, and collective action across perioperative pathway development.

## Introduction

Unwarranted clinical variation represents a significant evidence-to-practice gap that signals underutilisation of effective care, overuse of low-value interventions, and potential patient harm [1, 2]. Care variation becomes unwarranted when it varies in ways that are not in response to available evidence or to the needs and informed choices of patients [3]. Variation in the surgical setting has drawn the attention of quality and safety efforts internationally [4–6]. Inter-surgeon variation in outcomes may arise because of several complex and interacting factors. For example, surgeons can work autonomously with limited peer interaction, using individual preferences alongside evidence to make care decisions [7–9]. This can result in multiple surgeon-specific protocols for the same procedure [10], leading to inconsistency in care quality. Other contributing factors may include differences in workflows and team structures, resource availability within different healthcare settings, organisational culture and individual surgeon training [6, 11, 12]. Pathways that standardise the surgical care of patients across the preoperative, intraoperative and postoperative periods, according to best available evidence are effective in improving patient outcomes while lowering costs and postoperative length of stay [13–15]. These pathways are widely used across surgical specialties including colorectal surgery, gastrointestinal surgery, cardiac surgery and caesarean delivery. Key elements that a pathway may cover include clinical actions such as preoperative counselling, optimisation of nutrition, standardised analgesic and anaesthetic regimens and early mobilisation [16–18]. Despite the large evidence base supporting their clinical efficacy [13, 14, 18–20], pathways are considered a complex intervention due to their multimodal nature, and implementing them in a sustained and consistent way remains challenging [21–23].

Here, we present a process evaluation for an effectiveness-implementation hybrid type 3 quasi-experimental pre-post study [24] which used targeted implementation strategies to develop and implement standardised perioperative pathways. Six perioperative pathways detailing key clinical actions to be completed across each phase of the patient surgical journey, were developed and implemented at a private hospital in Australia using an organisationally-supported, multifaceted implementation plan [25]. Guided by the Exploration, Preparation, Implementation and Sustainment (EPIS) framework [26], which is a determinant framework that outlines four key phases of the implementation process, the implementation package incorporated multiple strategies, including informal local consensus discussions, which was the central component to develop each pathway involving multidisciplinary teams to tailor pathways to the local context, complemented by leadership engagement [27, 28]. This differed from more formalised consensus approaches, such as the Delphi method, or implementing an established pathway such as Enhanced Recovery After Surgery (ERAS) protocols [29], without local adaptation.

While there remains a need for more experimental trials that test implementation strategies against control conditions [30], researchers are often engaged to partner on non-randomised local implementation efforts [31], as was the case for this study. In such circumstances, control conditions are often not feasible, and these studies are often constrained by risks to internal validity through selection bias and confounding [32]. However, these projects offer critical opportunities for the field to learn about implementation in context; grounded in the realities of frontline care delivery and how strategies are adapted in real time to respond to prevailing conditions and achieve success [30, 31].

Systematic, theory-informed process evaluations are integral to maximising the external validity of such single-site quasi-experimental studies [33, 34]. By examining empirical data through a theoretical lens, researchers can better understand how different agents and factors interact through underlying mechanisms [35]. This approach supports the examination of the effects of implementation plans and their constituent strategies, helping to disentangle the specific contributions of each to overall implementation. A case in point is local consensus discussions; the primary strategy in our implementation package. While a useful strategy to develop clinical interventions and implement evidence into healthcare settings [36], it commonly co-occurs with other strategies as part of multifaceted bundles (here it was supported by other strategies) which can make it difficult to identify the effects of consensus discussions in isolation [36, 37]. Consensus discussions are assumed to improve the uptake and performance of evidence-based interventions when used to develop buy-in [37, 38]. Yet, the evidence remains inconclusive as to whether these effects are attributable to the consensus process itself, or the combined influence of an implementation bundle [37]. The Consensus Model for Standardising Healthcare demonstrates how clinicians navigate consensus-building in complex settings through evidence-seeking, a shared purpose, and structured deliberation to achieve agreement [10]. But examining implementation outcomes and applying theory can further identify the mechanisms through which consensus discussions work to affect sustainable change [35] and enable better design and adaptation of the strategy [39, 40].

Our study provides an example of a pragmatic, theory-informed process evaluation of an organisationally-supported consensus approach to developing and implementing standardised perioperative pathways. Specifically, the study aimed to:

1. Assess key implementation outcomes including acceptability, feasibility, and appropriateness of our multifaceted implementation package to implement standardised perioperative pathways, guided primarily by local consensus discussions, and examine the resultant fidelity to the intervention.
2. Provide practical insights on conducting a theory-informed process evaluation that can generate transferable insights for implementation science.
3. Explore stakeholders’ experiences of the implementation, including barriers and enablers.

## Methods

A convergent multi-method [41] effectiveness-implementation Hybrid (Type 3) pre–post study was conducted between October 2022 and December 2024, using participant observation, semi-structured interviews and a medical record audit. Full methodological details have been described elsewhere [25, 42, 43]; here, we provide a concise summary of the key steps to aid interpretation. A quasi-experimental design was utilised as the investigator team had no control over surgical cohort participation or the timing of pathway implementation. An observational approach was appropriate given our aim to study the process of implementation as it naturally unfolded in the real-world [44]. Ethical approval was received from the Macquarie University Human Research Ethics Medical Sciences Committee (Reference No: 520221219542374). The manuscript follows the Standards for Reporting Implementation Studies (StaRI) guidelines [45] (Additional File 1).

### Settings and implementation

The study was conducted in one university-owned, private teaching hospital located in metropolitan Sydney, Australia. The hospital comprises 144 beds, 16 operating theatres and is staffed by over 200 health professionals. Standardised perioperative pathways were the target intervention for this study. Pathways for four surgeries s (total hip arthroplasty (THA), total knee arthroplasty (TKA), radical prostatectomy and spinal) were initially included. Following pathway development for the first four surgeries, two additional surgeries (breast cancer surgery, percutaneous coronary intervention) were included. The surgical cohorts (i.e., the surgery, including its procedural elements, patients undergoing that procedure and the multidisciplinary teams responsible for their perioperative care) represented a heterogenous sample within the hospital where healthcare teams generally operate on separate wards in distinct teams, with limited cross-specialty interaction. Consequently, implementing each pathway required the coordinated implementation of multiple evidence-based clinical actions across different clinical stages, physical spaces, teams and specialties.

Implementation of the perioperative pathways followed a four-step process nested within four implementation phases using the EPIS framework [26]. A clinician–researcher facilitated this process, supported by hospital leadership and the external research team. To develop each pathway, a list of individual clinical actions spanning the perioperative continuum were identified. Individual pathway components represent evidence-based clinical actions that are recognised as being part of routine hospital care but may have been implemented inconsistently prior to pathway introduction. As such, individual clinical actions were drawn from multiple sources including evidence-based care guidelines, ERAS protocols [29], and/or current practice. Accordingly, the pathway functioned as a structured clinical tool to support more consistent and reliable adherence to these actions. Following identification of clinical actions, cohort-specific consensus groups, comprising clinical, non-clinical, and leadership representatives, participated in a series of regular informal consensus discussions to review these actions, propose additional items, and determine which should be included in a standardised perioperative pathway. As consensus-building is inherently iterative and depends on the availability and input of multiple clinicians, the number of meetings required varied across cohorts. Discussions continued until consensus was determined to be reached.

Then, draft pathways were disseminated to surgeons across the broader specialty for review and feedback prior to finalisation. These meetings also supported the co-development of a multifaceted implementation and evaluation plan with clinicians, stakeholders and implementation science experts, incorporating various strategies according to the Expert Recommendations for Implementing Change taxonomy (ERIC) [46] to facilitate each stage of the project (See Table 1 for full details). The clinician–researcher met with key clinicians and staff, such as nurse unit managers and physiotherapy leads, within each cohort who were identified as able to support deployment of the implementation plan. The taskforce (convened in the Exploration Phase) met monthly to identify actions that may be needed to support implementation on an ongoing basis.

**Table 1:** Initial Co-designed Implementation Blueprint

Implementation stage and timeframe^1^	Implementation strategy^2^	Description of phase purpose
*Phase 1: Exploration* *Projected timeframe: months 1—4*	Build a coalition	Establish a care pathway support team (taskforce) to convene monthly to proactively identify and address clinician and patient needs for optimal implementation
*Phase 2: Preparation* *Projected timeframe: months 4–12*	Conduct local consensus discussions	Establish cohort-specific clinical consensus groups with multidisciplinary representation to develop consensus on perioperative pathway components^2^ and a formal implementation blueprint. Groups to attend regular meetings led by an internal facilitator to establish consensus through informal consensus methods
	Co-develop a formal implementation blueprint	
*Phase 3: Implementation* *Projected timeframe: months 4–12*	Develop and implement care pathway toolkits	Implement perioperative pathways using formal implementation blueprints. Develop and introduce quality improvement tools and educational materials for clinicians to support implementation. Embed a system audit around care variation, clinical and process outcomes and disseminate findings to clinicians and administrators to monitor, evaluate, and modify provider behaviour
	Audit and provide feedback	
*Phase 4: Sustainment* *Projected timeframe: months 24-ongoing*	Facilitate relay of clinical data to providers	Continued application of the structures and processes to integrate pathways into routine practice. Continue formal monitoring and evaluation. Develop a reporting structure and channels of communication for ongoing care pathway development, implementation, and outcomes

Table adapted from the publication of the protocol for this study [15]

^1^Timeframes specified represent an approximate guide based on the earliest surgical cohorts to commence pathway design and implementation. As pathways were developed in a staged manner, some overlap occurred and timeframes varied as additional pathways were introduced at different stages. 2 Strategies informed by ERIC Taxonomy [33], ^2^Perioperative pathways were informed by ERAS principles [16] and other evidence-based guidelines, however were developed from the ‘ground up’, where evidence-based elements were modified as required to ensure the pathways were acceptable to each discipline and adaptable to local contexts

### Participants and recruitment

All local hospital staff from clinical, non-clinical and leadership roles involved in clinical consensus groups to develop each pathway or those involved in pathway implementation were considered eligible for inclusion. No specific exclusion criteria were applied. Eligible staff were approached to participate in an interview and/or observation during the Preparation and Implementation phases using a convenience sampling approach. Participants were provided with an information form prior to providing verbal or written consent. Consent was obtained prior to participation. Participants could withdraw their consent or opt-out at any time during data collection.

### Data collection

#### Qualitative data

An interpretive descriptive approach was used [47]. Naturalistic participant observations of consensus or implementation planning meetings were conducted by an experienced field researcher (LP). Field notes were recorded in real-time, with detailed notes completed after each observation. Observations were not guided by a pre-determined schedule, allowing ideas to emerge inductively.

Face-to-face semi-structured interviews were conducted by the field researcher (LP) to supplement participant observations. Interviews took ~ 30–60 min and followed purpose-designed topic guides (Additional File 2), drawing on principles from existing literature relating to consensus processes, implementation science, decision-making, organisational and behaviour change (key references include: [26, 48–55] and the research teams’ implementation experience. Topic guides were piloted prior to use with two clinicians from the organisation and modifications were made as required. Questions were open-ended with flexibility in their order and wording, and probes were used to clarify statements. Field notes and conceptual memos including initial thoughts, interpretations and data analyses were recorded after each interview and throughout analysis. Interview transcripts were digitally recorded and transcribed verbatim. An audit trail of methodological decisions made throughout the study were recorded. Data collection and analysis occurred concurrently, and data collection was discontinued when data saturation had been achieved [56].

#### Quantitative data—patient medical record audits

A medical record audit was conducted 12 months post implementation to determine clinician adherence to the pathways and if their implementation improved compliance to recommended care guidelines. A random sample of patients who had undergone surgery from one of the four initial surgical cohorts (THA, TKA, radical prostatectomy or spinal surgery) to enable sufficient data for pre/post comparison, were selected for audit via the electronic medical records system Trackcare. For each cohort, 30 patients who had surgery during a three-month period (February, March, April) in the years 2022, 2023 and 2024 were randomly selected using the Excel random number generator tool, totalling 90 cases per cohort. Across these sampling periods, the total number of eligible patients per cohort were as follows: THA = 193 patients (2022 *n* = 75, 2023 *n* = 59, 2024 *n* = 59); TKA = 247 patients (2022 *n* = 92, 2023 *n* = 75, 2024 *n* = 80); Spinal = 116 patients, (2022 *n* = 42, 2023 *n* = 43, 2024 *n* = 31) and radical prostatectomy = 185 patients (2022 *n* = 73, 2023 *n* = 64, 2024 *n* = 48).

Specific clinical actions within each pathway were selected as audit data points by a clinician-researcher (AH) in consultation with clinical interest-holders (see Table S1 for complete list). Data points were extracted from Trackcare into a purpose-designed REDCap survey instrument. For a quality assurance process, a total of 5% of included records (*n* = 5) were extracted by two research students and two researchers (AH, LP) from the radical prostatectomy cohort. A further 10% of records (*n* = 9) from each cohort were extracted by each student and checked by either AH, LP or the other student for completeness and correctness. Any conflicts were resolved by AH. The remaining records were then extracted by one of two research students under the supervision of AH and LP.

### Implementation outcomes

Proctor et al.’s (2011) implementation outcomes of acceptability, adoption, appropriateness, feasibility and perceptions of sustainability [46] were assessed using data from qualitative observations and interviews. Quantitative data via medical record audits were used to assess fidelity post implementation. Implementation fidelity was defined using Carroll and colleagues (2007) framework as intervention adherence (i.e., the degree to which those responsible for delivering the intervention adhered to the intervention as outlined by its designers) [57]. Adherence was determined by content, frequency and duration (i.e., dosage) by calculating the aggregate score of the total number of clinical actions within each pathway received per patient, out of the total number of intended elements (target outcome). In addition, we examined comparative process performance pre (2022) and post (2024) implementation, to determine if introducing the pathways improved uptake of recommended evidence-based clinical actions in each pathway. This was determined from the number and occurrence of individual clinical actions within each pathway received per patient. Since there were no changes to the eligibility of patients to receive care under the new pathways, coverage or population penetration was expected to remain at 100% of the pre-implementation baseline. Qualitative data were also used to identify potential moderators and modifications to the implementation strategy that could influence fidelity.

### Data analysis

#### Qualitative analysis

Transcripts and observation field notes were analysed using NVivo, V14. Analysis of primary implementation outcomes followed a multi-stage approach. In Stage 1, interview transcripts and observation field notes were analysed using reflexive thematic analysis methods, described by Braun and Clarke which emphasises researcher reflexivity and interpretive engagement with the data [58–61]. Transcripts and field notes were thoroughly read to ensure immersion. To develop the initial coding framework, inductive, line-by-line coding was conducted with constant comparison to other data segments and codes on two transcripts and one field note by LP. Initial codes were then discussed with two qualitative researchers (JL, MNS). LP then coded the remaining transcripts, field notes and conceptual memos, with team support and peer-debriefing with JL, EF-A and MNS. The most significant codes were synthesised into meta-codes and categories to identify emerging concepts with agreement by LP, JL, EF-A and MNS. Interview guides for subsequent interviews were then refined to include more focussed questions, and initial interpretations and core concepts were tested in subsequent interviews and observations. Data from observations and interviews were triangulated. Themes were developed based on meta-codes and patterns of meaning within the data and discussed and agreed upon by the team. Proposed themes were also discussed with the clinician-researcher (AH) who facilitated the consensus process and each observation to support interpretive sense-checking and ensure the analysis reflected clinical and contextual insights.

In Stage 2, emergent Stage 1 themes were further examined to understand their relationships to EPIS constructs [26]. These themes were organised and deductively allocated to one of the four EPIS constructs (Outer context, Inner context, Bridging factors, Innovation factors) and elements within each construct by LP. An analytical summary matrix was developed to organise the domains and themes. LP then examined how EPIS constructs and elements captured in the themes related to implementation outcomes [46]. Themes were mapped to Proctor et al.’s (2011) outcomes to explore how participants viewed each theme and how they were relevant to or influenced an implementation outcome. MNS was consulted in team meetings to check consistency and coherence of the proposed allocations.

In Stage 3, we identified hypothesised causal mechanisms by which the initial implementation plan (Table 1) worked to promote intervention uptake and sustainability. Normalisation Process Theory (NPT) was used to support this analysis since it provides a structured, theory-informed framework, widely applied in implementation research to examine how an intervention becomes normalised into practice [62, 63]. Interview and observation data related to the initial implementation plan and perceptions of how implementation occurred were re-analysed, considering; i) which strategies were perceived as successful to support implementation; ii) factors that influenced or facilitated progress through each EPIS phase and iii) additional ERIC strategies that were adopted by the organisation or identified as valuable for future implementation efforts. Stage 1 codes describing strategies were mapped to relevant ERIC strategies and EPIS phases within the initial plan by LP. Drawing on NPT, we explored the underlying mechanisms that shaped how each implementation phase functioned (or could function), contributing to both successful implementation and integration of pathways into routine care. ‘Conducting local consensus discussions’ was examined in detail as a case example to illustrate how an individual implementation strategy may function to influence implementation. For Stage’s 2 and 3, regular team meetings and peer debriefing sessions were held to enhance reflexivity, interrogate emerging interpretations, refine allocations, and strengthen the credibility and depth of the developing analysis.

#### Quantitative analysis

Quantitative fidelity data were extracted and imported into IBM Statistics Package for Social Sciences (SPSS) Version 29.0. The full analysis plan is in Additional File 3. In brief, for each pathway element, adherence to the planned dosage was assessed as the percentage of patients who received compliant care. Each audited clinical action was first scored as 0 for non-compliance or 1 for compliance and then summed for each patient per audit year to derive a patient compliance score. Where data were missing, the action was considered not documented and subsequently classified as non-compliant. Descriptive statistics were calculated to describe the distribution of individual patient pathway compliance scores (mean [SD], median [IQR], minimum, maximum) by cohort and year. The distribution of scores for the target outcome were assessed as non-parametric using both visual inspection and statistical tests of non-normality.

The primary analysis was specified as a two-sample t-test, comparing the pre- and post-intervention pathway scores, as the t-test is robust to deviations from normality [64]. The primary analysis compared 2022 (pre-intervention) with 2024 (post-intervention); 2023 was considered a transition year to indicate whether pathway component compliance had been anticipated by practice. Planned post-hoc analyses were conducted to explore which clinical actions contributed to statistically significant changes in the primary analysis using Fishers exact test (*p* < 0.05).

Secondary analyses adjusted for the impact of potential confounding of baseline clinical risk in each cohort: age (all cohorts); sex (all cohorts except prostate surgery); and body mass index (BMI) (THA and TKA only). Potential confounding variables were purposefully screened and selected by assessing their maldistribution between the pre- and post-intervention periods and/or causal link as a risk factor for pathway adherence score. Variables were considered maldistributed where there was a difference of 10 percentage points (absolute change in binary variables) or a 10% relative difference in median (for continuous variables) between pre- and post-intervention periods. Univariable median quantile regression was then conducted across all cohorts (2022–24) to test if there was any link between potential confounders and pathway score (i.e. predictive of the outcome) [65]. Continuous variables were first broken into quartiles and the average pathway score for each quartile was calculated to determine the best shape to include in the model (e.g., linear, quadratic, or categorical explanatory variable). A threshold of *p* < 0.2 indicated a potential risk-factor status. Bivariable models were fit by adding each potential confounder, comparing 2022 and 2024 pathway scores. If the estimate of effect (post- vs pre-intervention) changed by 10% (relative change) and a minimum of 0.5 points (absolute score change), the potential confounder was retained [66]. Where two or more confounders were retained in bivariable analysis, each additional variable needed to result in a 10% relative change (and 0.5-point absolute change) for that variable to be retained in the final multivariable models [66].

## Results

### Qualitative findings

All invited observation opportunities were attended by LP, which comprised 15 consensus discussions (approximately 16 h of observation) with 31 staff. All staff present at the meetings consented. Seventeen staff members were invited to participate in semi-structured interviews of which two declined, and seven did not respond. Nine interviews were completed (one participant was interviewed twice for theoretical validation purposes). Participant characteristics and observation details are in Table 2.

**Table 2:** Characteristics of observation and interview participants

Item	Number (%)
***Interview participants***	***n* = 8**^1^
**Discipline**	
Leadership/management	4 (50%)
Registered nurse	2 (25%)
Anaesthetist	1 (13%)
Physiotherapist	1 (13%)
**Gender**	
Female	6 (75%)
Male	2 (25%)
**Number of years worked at organisation**	
< 5 years	3 (38%)
6–10 years	4 (50%)
11 + years	1 (13%)
**Number of years worked in healthcare**	
10–20 years	3 (38%)
20–30 years	2 (25%)
30–40 years	2 (25%)
40 + years	1 (13%)
**Observation sources**	
***Meeting type***	***n* = 15**
**Consensus meeting for pathway development and/or implementation**	
Spinal surgery	5 (33%)
Total hip and knee arthroplasty	3 (20%)
Percutaneous coronary intervention	3 (20%)
Breast cancer surgery	2 (13%)
**Nursing committee and/or leadership meetings**	2 (13%)
*** Observation participants***	***n* = *31***^***2***^
Registered nurse	10 (32%)
Surgeon	7 (23%)
Nursing unit manager	6 (19%)
Leadership/management	4 (13%)
Anaesthetist	2 (6%)
Physiotherapist	2 (6%)

^1^A total of nine interviews were completed with one participant interviewed on two occasions; ^2^22 participants observed on more than one occasion

**Fig. 1:**
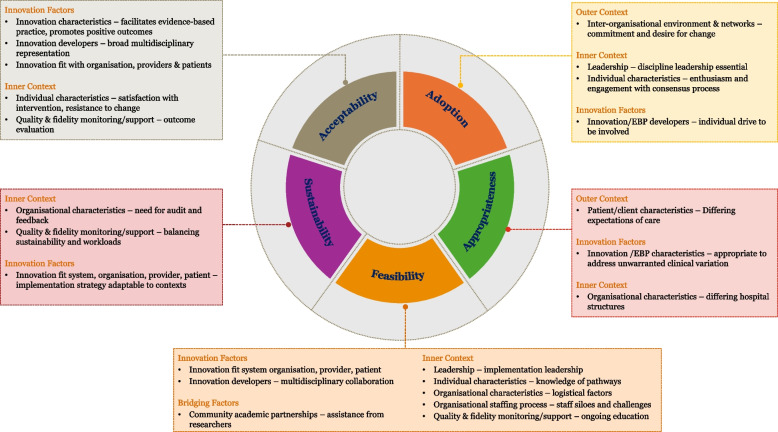
Visual representation of the EPIS constructs mapped to Proctor et al.’s (2011) implementation outcomes framework including a summary of emergent themes related to each implementation outcome and EPIS construct (Moullin et al., 2019)

Themes that influenced implementation of the pathways related to all four constructs of the EPIS framework and 12/16 elements within the constructs. Factors related to the Inner Context and the Innovation were the most frequently identified and appeared to have greater influence on implementation across all EPIS phases. Below we summarise the key findings of each implementation outcome relating to the perioperative pathways and the consensus development process (i.e., the implementation strategy). Figure 1 presents the EPIS constructs mapped to Proctor et al.’s implementation outcomes with a summary of emergent themes. Table S2 contains the complete list of emergent themes and exemplar quotes from the included studies.

### Implementation outcomes

#### Acceptability

The development of standardised pathways was perceived as acceptable among staff involved in both pathway development and implementation. Participants recognised the value of standardisation as an opportunity to improve different hospital care processes. Many aspects of the implementation plan used to develop and implement the pathways were considered acceptable, particularly the opportunities created for multidisciplinary input during consensus discussions. Broad representation within surgical disciplines was viewed as helpful for promoting uptake by making the pathway more palatable to other clinicians. However, participants from nursing and allied health noted limited inclusion of frontline clinicians and over-representation of leadership and mid-level management in consensus groups. Involving frontline clinicians in pathway development was key to improving pathway fidelity and understanding downstream effects. Factors within the Inner Context influenced clinicians’ willingness to change their behaviour, with some more hesitant to change extant practices e.g., surgeons valued professional autonomy, preferring flexibility to use their clinical judgement alongside standardised pathways.

#### Adoption

Factors within two domains were found to influence adoption of the pathways and implementation strategy. In the Outer Context, the organisations’ desire to align with peer organisations on key performance metrics (‘inter-organisational environment and networks’) motivated the project. Consequently, the organisation was ready and willing to commit time and resources to this initiative. In the Inner Context, leadership at all levels was key to promoting clinician adoption. Surgical discipline leads were critical in endorsing each pathway, especially within their own disciplines. Leadership from nursing and allied health was valuable to increase pathway visibility and adoption on the wards for clinicians not involved in pathway development. While offering incentives, such as the prospect to collect outcomes to guide clinicians’ work was helpful, staff reported that individual motivation and belief in the value of standardisation were the strongest drivers of engagement.

#### Appropriateness

Implementing standardised pathways was considered appropriate by many participants to reduce unwarranted variation in care, largely due to aspects within the Innovation Factors domain. Standardised pathways were seen as compatible with the organisations’ desire to streamline care processes, reduce confusion among staff and reduce variation arising from having individual surgeon pathways, especially by nursing and allied health. However, questions over compatibility of the intervention within the private hospital setting were raised due to the different medical discipline structures compared to public hospitals. In private settings, regular interdisciplinary interactions may be limited, posing challenges for achieving consensus, gaining buy-in and maintaining adherence to standardised practices.

#### Feasibility

Feasibility depended on multiple factors relating to Innovation Factors and the Inner Context of the healthcare setting. Relating to the Innovation, adaptability of the implementation strategy was crucial. Implementation blueprints developed during the Exploration Phase were based on insights from previous quality improvement activities completed in the orthopaedic discipline. These required tailoring during the Implementation Phase to suit the diverse working practices of different disciplines to ensure a better fit within existing workflows. Involving frontline staff supported adaptation, though limited multidisciplinary representation in some meetings and competing clinical demands slowed implementation progress by increasing the number of discussions required to reach consensus. Organisational Characteristics, particularly staffing structures (e.g. siloed practices that limit interdisciplinary collaboration) and limited resource availability, negatively impacted feasibility where participants noted increased workload, communication gaps, and inadequate technology systems. However, research partnerships were seen as helpful for designing evaluation processes.

#### Sustainability

Staff identified key elements affecting the long-term viability of the pathways, largely centred around the Inner Context. The need for ongoing staff education modules in addition to one-off onboarding modules was highlighted as key to continuously reinforcing desired clinical behaviours. Additionally, ‘Quality fidelity and monitoring’ could provide a structured mechanism to reinforce pathway awareness through feedback processes. During the study period, a one-off iterative audit and feedback process was introduced with findings shared with surgeons and discipline leaders. Staff suggested this process should be conducted more regularly and feedback delivered to all clinicians involved. However, concerns were raised about the time and workload required to collect data, highlighting the need for additional resources and better technological support.

### Quantitative findings—fidelity

#### Primary analysis

Fidelity to each pathway following implementation is reported in Table 3. Process improvements to the total number of recommended clinical actions received per patient from 2022 to 2024 were observed in two of four surgical cohorts: THA (2022 = 11.27,2024 = 12.53, mean difference 1.26 points, *p* = 0.002) and TKA (2022 = 11.57,2024 = 12.60, mean difference 1.03 points, *p* = 0.006). Pathway implementation had non-significant effects for the radical prostatectomy cohort (2022 = 9.17,2024 = 9.53, mean difference 0.38 points, *p* = 0.2) and the spinal surgery cohort (2022 = 6.20,2024 = 5.93, mean difference −0.27 points, *p* = 0.4).

**Table 3:** Compliance to individual clinical actions

Pathway variable	THA			TKA			Radical prostatectomy			Spinal surgery		
	**2022**	**2024**	***p*-value**	**2022**	**2024**	*p***-value**	**2022**	**2024**	**p-value**	**2022**	**2024**	*p***-value**
Before surgical admission												
Attended PAC	26 (87%)	27 (90%)	*p* = 1.00	26 (87%)	29 (97%)	*p* = 0.35						
RAPT form completed	14 (47%)	23 (77%)	*p* **= 0.03**	16 (53%)	9 (30%)	*p* = 0.12						
PAC attendance within 3–4 weeks of day of surgery	25 (83%)	24 (80%)	*p* = 0.92	24 (80%)	24 (80%)	nc						
Physiotherapy review at PAC appointment	23 (77%)	26 (87%)	*p* = 0.51	22 (73%)	28 (93%)	*p* = 0.08						
Patient health questionnaire completed	30 (100%)	28 (93%)	*p* = 0.49	27 (90%)	30 (100%)	*p* = 0.24	30 (100%)	28 (93%)	nc	27 (90%)	25 (89%)	nc
During surgical admission												
Admissions tab completed	18 (60%)	22 (73%)	*p* = 0.41	17 (57%)	22 (73%)	*p* = 0.28	24 (80%)	22 (73%)	nc	22 (73%)	25 (89%)	nc
Admitted on day of surgery	24 (80%)	28 (93%)	*p* = 0.25	28 (93%)	30 (100%)	*p* = 0.49	30 (100%)	30 (100%)	nc	25 (83%)	20 (71%)	nc
VTE assessment completed on day of surgery	29 (97%)	30 (100%)	*p* = 1.00	30 (100%)	30 (100%)	nc	29 (97%)	29 (97%)	nc	28 (93%)	28 (100%)	nc
VTE compliance^1^	23 (77%)	27 (90%)	*p* = 0.30	24 (80%)	28 (93%)	*p* = 0.25	26 (87%)	28 (93%)	nc	24 (80%)	16 (57%)	nc
Planned discharge destination identified preoperatively	26 (87%)	27 (90%)	*p* = 1.00	25 (83%)	25 (83%)	nc						
SAP according to guidelines	16 (53%)	28 (93%)	***p*** **< 0.001**	23 (77%)	28 (93%)	*p* = 0.15						
Mobility compliance^2^	8 (27%)	7 (23%)	*p* = 1.00	5 (17%)	9 (30%)	*p* = 0.36	28 (93%)	30 (100%)	nc	30 (100%)	28 (100%)	nc
IDC compliance^3^	14 (47%)	22 (73%)	*p* = 0.06	18 (60%)	29 (97%)	***p* = 0.001**	30 (100%)	30 (100%)	nc			
Patient controlled analgesia compliance^4^	30 (100%)	29 (97%)	*p* = 1.00	27 (90%)	30 (100%)	*p* = 0.24						
CNC review during admission							30 (100%)	30 (100%)	nc			
Discharge summary received by date of discharge	26 (87%)	20 (67%)	*p* = 0.13	23 (77%)	24 (80%)	*p* = 1.00	16 (53%)	21 (70%)	nc	26 (87%)	24 (86%)	nc
After surgical admission												
CNC review within 2 weeks of discharge							7 (23%)	12 (40%)	nc			
Prostate cancer nurse review within 1 month of discharge							25 (83%)	26 (87%)	nc			

Statistically significant improvements in fidelity to indicators from pre to post implementation are bolded

*CNC* Clinical nurse consultant, *IDC* indwelling catheter, *nc* not calculable, *PAC* preadmission clinic, *Preop* preoperatively, *POD* postoperative day, *RAPT* Risk Assessment and Prediction Tool, *THA* total hip arthroplasty, *TKA* total knee arthroplasty, *SAP* surgical antibiotic prophylaxis, *VTE* venous thromboembolism

^1^Compliant if patient was prescribed both Thrombo-Embolic Deterrent stockings & Sequential Compression Devices; ^2^For THA/TKA, compliant if patient was mobilised on Post-operative Day 0. For spinal surgery/prostatectomy, compliant if patient mobilised on Post-operative Day 0 or 1; ^3^For THA/TKA, compliant if patient did not have an IDC or if IDC was removed within 24 h of surgery. For prostatectomy, compliant if IDC was changed to a leg bag prior to day of hospital discharge; ^4^Compliant if patient did not have a patient-controlled analgesia or if patient-controlled analgesia was removed within 48 h of surgery.Indicator for 0.05, < 0.01, < 0.001

Following pathway implementation, 10/15 and 11/15 individual clinical actions showed higher compliance rates for THA and TKA cohorts respectively (Table 4). For radical prostatectomy, 5/11 showed higher compliance rates, and for spinal surgery only 2/7 showed higher compliance rates. In these cohorts, baseline rates of compliance were high for most variables, being 80% or over for 9/11 prostatectomy variables and 6/7 spinal surgery variables.

**Table 4:** Baseline compliance scores and number of indicators that increased post-intervention

Baseline compliance rate	THA		TKA		Prostatectomy		Spinal	
	No. of indicators	No. (%) Increased	No. of indicators	No. (%) Increased	No. of indicators	No. (%) Increased	No. of indicators	No. (%) Increased
< 60%	4	3 (75%)	3	3 (100%)	2	2 (100%)	0	-
60–79%	3	3 (100%)	4	4 (100%)	0	-	1	1 (100%)
≥ 80%	8	4 (50%)	8	5 (63%)	9	3 (33%)	6	1 (17%)
ALL	15	10 (67%)	15	12 (80%)	11	5 (45%)	7	2 (29%)

*No.* number, *THA* total hip arthroplasty, *TKA* total knee arthroplasty

A modest number of clinical actions showed statistically significant changes within pathways that changed significantly (Table 3). For the THA cohort, surgical antibiotic prophylaxis received according to guidelines (pre 53% vs post 93%, *p* < 0.001) and Risk Assessment and Prediction Tool (RAPT) completion (pre 47% v post 77%, *p* = 0.03) changed significantly; in light of the small sample size we note that indwelling catheter compliance appeared higher in the THA cohort (47% vs 73%), but not significantly so (*p* = 0.06). For the TKA cohort, only indwelling catheter compliance (pre 60% v post 97%, *p* = 0.001) significantly increased. Physiotherapy review at pre-admission clinic appointment increased from 73 to 93% however, this was below statistical significance (p = 0.08). RAPT form completion decreased in the TKA cohort (pre 53% vs post 30%, *p* = 0.12) despite both pathways being developed by the same consensus groups and largely implemented by the same group of clinicians (and increasing for THA).

#### Secondary analysis

Both sex and BMI were identified as confounders for the THA cohort. After adjustment, the median difference in compliance scores increased from 2.0 to 2.3. The median post-intervention score was statistically significant in both the univariable and final multivariable models (*p* = < 0.001 and *p* = 0.001 respectively). In both the TKA and spinal cohorts, BMI and age were identified as potential confounders respectively, however, the difference in median score was 0 in both the univariable analysis and the final model. Of the potential confounders for which data were available, none were identified as associated with adherence scores for the radical prostatectomy cohort.

### Fidelity

#### Overall implementation plan

All implementation strategies included in the initial implementation blueprint (Table 1) were utilised and considered useful by staff involved in the implementation effort. However, it became clear as implementation progressed through each EPIS phase, that additional strategies were needed to support integration of the intervention into routine practice. Supplementary strategies were iteratively adopted by the organisation in response to emerging barriers however, participants identified additional strategies that would be beneficial to include in future efforts.

NPT helped to identify hypothesised mechanistic pathways driving implementation outcomes not captured in quantitative analyses and why additional strategies were needed to enhance sustainability (Table 5). Implementation was operationalised through the four generative mechanisms of NPT: Coherence, Cognitive Participation, Collective Action, and Reflexive Monitoring [63]. Coherence (i.e., how participants made sense of the intervention) was well-established during the Exploration and Preparation phases among staff in consensus groups, however, further strategies that communicated meaning and utility of the pathways to frontline clinicians were needed in subsequent phases. Cognitive Participation (i.e., commitment and engagement by participants) was promoted through local consensus discussions. However, legitimation (i.e., buying into a practice) could have been further strengthened by additional strategies, such as greater multidisciplinary representation in consensus discussions and identifying implementation champions from all levels of leadership and various disciplines.

**Table 5:** Updated multifaceted implementation plan informed by Normalisation process theory

EPIS phase	NPT construct	Implementation strategy^1^	Description from protocol [15]	How the strategy worked in practice and additional lessons
*Exploration*	Coherence	Build a coalition	Recruit and cultivate relationships with partners in the implementation effort by formally establishing a care pathway support team	**How it worked**: One clinician researcher internal to the organisation, in collaboration with researchers and hospital leadership, established a ‘coalition’ which functioned as a strategic advisory and governance committee. The group met monthly to review progress and actions needed to support implementation. Members included hospital management, quality and safety representatives, researchers, and clinical representatives (medicine, nursing and allied health) **Additional lessons**: Including frontline care providers in the coalition could enhance frontline clinician adoption, by ensuring their perspectives inform decision-making
		*Additional strategies applied & not defined in initial implementation plan*		
	Collective action (interactional workability)	Develop academic partnerships	Partnering with an academic institution during the exploration stage proved valuable to help establish procedures and develop an evaluation framework with a focus on long-term sustainment	
		*Strategies to consider in future implementation plan*^*2*^		
	Collective action	Assess for readiness and identify barriers and facilitators	Barriers and facilitators to implementing pathways for different cohorts were identified during the development and implementation phases, with solutions applied iteratively and informally shared with future cohorts. A more formalised approach to assessing readiness could better identify needs, strengths, and optimal timing for implementation	
*Preparation*	Coherence, Cognitive participation (initiation, enrolment); Collective action (interactional workability, relational integration)	Conduct local consensus discussions	Establish a structure for local providers and other stakeholders to form cohort-specific clinical consensus groups to discuss the processes of care and standardisation of pathways	**How it worked in practice:** the consensus process was adapted to suit the needs of different cohorts: i) cohort specific groups met face to face, ii) meetings divided amongst smaller discipline specific groups (e.g. nursing, medical) held separate discussions and, iii) one discipline lead developed consensus with leadership before sharing for broader feedback **Additional lessons:** Multidisciplinary involvement is crucial. Adapting the process of consensus to suit each cohort’s needs and team dynamics promotes engagement and progress
	Collective action (interactional workability, relational integration)	Co-develop a formal implementation blueprint	Co-develop a formal blueprint for iterative care pathway prioritisation and implementation	**How it worked in practice:** a formal blueprint was developed through regular meetings with consensus groups and the care pathway support team. Findings were synthesised by the facilitator
		*Strategies to consider in future implementation plans*^*2*^		
	Cognitive participation (initiation, legitimation); Collective action (relational integration)	Identify and prepare champions	Utilising champions internal to the organisation from different clinical disciplines across hospital managerial, medical, and nursing can enhance credibility, improve early adoption and facilitate communication, ensuring broader awareness and smoother integration of the pathways	
*Implementation*	Collective action (interactional workability, skill-set workability	Develop and implement care pathway toolkits (develop educational materials)	Develop, test, and introduce quality improvement tools and educational materials	**How it worked in practice:** A dedicated education team developed pathway-specific educational materials for patients and frontline care staff **Additional lessons:** Removing the burden of resource creation from frontline clinicians was beneficial, but the necessity of materials for all disciplines should be assessed. A clear dissemination plan should be included as part of the toolkit. Consider documenting the key lessons and process of pathway development and consensus-building
	Cognitive participation; Reflexive monitoring	Audit and provide feedback	Embed a comprehensive system audit around care variation, clinical and process outcomes over specified time periods and disseminate to clinicians and administrators to monitor provider behaviour	**How it worked in practice:** A one-off documentation audit was completed with the assistance of researchers and students. Data presented to each discipline **Additional lessons:** Ongoing auditing requires the implementation of a regular monitoring system to reduce manual workload for clinical staff e.g. assigning audits to a designated quality improvement team. Feedback should be shared with all relevant staff
		*Strategies to consider in future implementation plans*^*2*^		
	Cognitive participation (enrolment)	Mandate change	Have leadership declare the innovation as a priority and their determination to have it implemented. Leadership mandates considered especially important for medical staff	
	Coherence; Cognitive participation (initiation); Collective action (interactional workability, skill-set workability	Educate staff and remind clinicians^3^	Utilising various strategies such as conduct educational meetings, conduct ongoing training and remind clinicians, targeted toward different clinical disciplines ensures clinicians are informed about the newly implemented pathways and any required practice changes. Ongoing reminders further reinforce these updates and maintain clinician engagement	
	Collective action (contextual integration); Reflexive monitoring	Tailor strategies and promote adaptability	Identify the ways the implementation strategy (e.g. how consensus discussions are held) can be tailored to meet the needs of each cohort. Identify and address barriers and facilitators and determine which elements of the strategy must be maintained to preserve fidelity and sustainability	
*Sustainment*	Reflexive monitoring	Facilitate relay of clinical data to providers	Undertake formal monitoring and evaluation and develop a reporting structure and channels of communication for care pathway development, implementation and outcomes	**How it worked in practice:** Consultations held with clinicians to determine which outcomes were meaningful to each cohort to examine change and adherence. Data then presented to surgeons and anaesthetists **Additional lessons:** Establish channels to report adherence data to nursing and allied health. Establish and integrate regular audits into practice to routinely monitor clinical processes. Additional ERIC strategies may be useful: Develop and organise quality monitoring systems, purposely reexamine the implementation
		*Additional strategies applied & not defined in initial implementation plan*		
	Collective action (contextual integration)	Capture and share local knowledge	**How it worked in practice:** The facilitator shared learnings with new cohorts during onboarding meetings, enabling them to adapt the process as needed	
		*Strategies to consider in future implementation plans*^*2*^		
	Cognitive participation (initiation, legitimation)	Alter incentive structures	Providing incentives, such as feedback on data and outcomes, can support the adoption and implementation of the clinical innovation by demonstrating its impact and reinforcing its value for clinicians	
	Collective action (interactional workability)	Centralise technical assistance	Centralise and build pathways within existing systems, such as EMRs, to reduce clinician workload and eliminate the need for separate outcome data entry	

Table presents the key theoretical constructs reflective within the implementation strategy identified as important to support the normalisation and integration of the intervention into routine practice. Shaded cells represent the strategies that were not included in the initial implementation blueprint and were either completed by the team or were identified in qualitative data collection as necessary to improve feasibility, adoption and sustainability of the intervention

*EMR* Electronic medical records, *ERIC* Expert Recommendations for Implementing Change

^1^Strategies and descriptions adapted from ERIC Taxonomy [33] ^2^Strategies to consider in future implementation plans as identified from qualitative data ^3^These strategies were also identified as essential for the sustainment phase of implementation

Collective Action (i.e., actions that make the intervention function) was achieved through different strategies, particularly using consensus discussions to co-design a plan to implement each pathway. Having an internal facilitator lead this process was essential, supporting interactional workability. While skill-set workability was generally unproblematic due to the alignment of practices with existing clinical remits, contextual integration posed challenges, highlighting the importance of adapting and tailoring strategies. Reflexive Monitoring (i.e., evaluation of an intervention) provided insights into implementation fidelity and it was recognised that ongoing monitoring and a centralised audit and feedback system to track pathway fidelity over time was needed.

#### Individual strategy – conducting local consensus discussions

Consensus discussions supported Coherence by building a shared purpose (communal specification), clarifying individual roles to support pathway implementation (individual specification), and understanding the intervention’s value (internalisation). Discussions promoted Cognitive Participation by facilitating the involvement of key individuals in decision-making (initiation), encouraging organisational engagement (enrolment), and strengthening participants’ belief in their contribution (legitimation). While consensus participants remained engaged throughout implementation phases (activation), further strategies were needed to sustain engagement among others not involved in pathway development. Consensus discussions supported preparation for Collective Action e.g. clarifying the division and delegation of tasks for implementation (skill-set workability) and establishing a clear plan of action moving forward.

## Discussion

This study demonstrates how meaningful theory-informed insights can be generated through clinician-led, real-world implementation efforts supported by implementation scientists when using established theories, models and frameworks. The perioperative pathways were generally perceived as acceptable, appropriate, and feasible; however, fidelity to key clinical actions varied, improving in only two of four surgical cohorts. Factors relating to the Inner Context and characteristics of the Innovation itself had the greatest influence on implementation across all EPIS phases. Utilising NPT enabled us to move beyond describing implementation outcomes alone, to better understand the potential mechanistic pathways underlying how the implementation plan worked and what could be improved**,** making our findings move beyond pure quality improvement.

This study highlights several practical lessons for clinicians seeking to conduct rigorous, theory-informed implementation evaluations. While identifying which implementation strategies are needed to facilitate implementation is valuable, knowing when to implement them is equally important. Selecting an implementation framework allowed us to map when certain strategies might be most effective during an implementation phase. For example, developing a training module was deemed a priority by consensus groups to foster Coherence and was incorporated into the early Implementation Phase in the implementation blueprint. Yet, a one-off training module proved insufficient to integrate and sustain the intervention where many clinical staff remained unaware of the pathways. Clinicians reported a need for more intensive and regular bouts of implementation training. Incorporating strategies previously found to be effective in improving professional knowledge and practice in guideline implementation, such as educational meetings and ongoing reminders [67], into the Implementation and Sustainment phases may have helped to bolster key mechanisms of Coherence, Cognitive Participation, and Collective Action. Incorporating EPIS alongside a theory like NPT, therefore, enabled us to identify which mechanisms were most relevant and to map future strategies to the appropriate time points within the implementation lifecycle.

Our findings suggest that local consensus discussions could function as a powerful implementation strategy to promote change, particularly when adapted to the dynamics of each cohort. Clinicians widely viewed the consensus process as acceptable and appropriate to develop perioperative pathways, likely due to the iterative and collaborative nature of consensus discussions. This approach potentially activated key NPT mechanisms theorised the support embedding and sustainment of innovations. We found consensus discussions appeared to work by fostering an understanding of the interventions’ purpose and value [68]. Yet, interestingly, while consensus-building literature states that a condition of consensus-building is the task must be inherently meaningful to participants [69], we found that the process of consensus-building itself helped generate shared meaning as the process progressed, which promoted acceptability. However, consensus-building alone was not sufficient to drive engagement, and visible leadership during meetings was needed to signal institutional commitment. Multidisciplinary representation and skilled facilitation were essential to navigating these discussions, consistent with Innes’ argument that one of consensus-building’s core functions is to surface diverse perspectives and clarify areas of uncertainty which likely promoted Collective Action [49, 69]. These findings align with prior research showing that both designated and distributed leadership are critical for system-wide change, with formal leaders playing a key role in fostering an organisational culture that enables greater clinician involvement [70–72]. Taken together, our study shows that informal consensus discussions can do more than secure agreement and may work to activate key mechanisms that support the normalisation of complex interventions into everyday practice.

The concept of implementation fidelity is important to measure since the extent to which an intervention is delivered as intended can impact its effectiveness [57]. Beyond theoretical importance, fidelity is feasible for clinicians to assess in real-world settings. While certain fidelity measures like dosage reveal whether an intervention was delivered as intended [57], examining moderators to fidelity could help us to understand how and why implementation strategies may succeed or struggle to promote fidelity. In our evaluation, we observed variability in fidelity across surgical cohorts pointing to several contributing factors. One was intervention complexity. Broader implementation literature suggests that complex interventions with a greater number of components, actors, and potential points of variation, are inherently more difficult to implement [53]. Despite developing an implementation blueprint that targeted different organisational levels to address this complexity, the observed variability suggests that certain mechanisms necessary for sustained fidelity were not sufficiently activated across all cohorts. Participant responsiveness also likely played a role [57] as clinicians involved in consensus groups generally demonstrated stronger engagement compared to frontline clinicians who were expected to implement the pathways. In hindsight, identifying and preparing implementation champions during the Preparation phase could have supported Collective Action, as exposure to champions has been associated with increased uptake of best practices [73–75]. Similarly, stronger leadership presence from each discipline may have improved fidelity, as effective leaders are known to maintain consistent engagement with quality improvement interventions [68, 75, 76]. It is important to note that we measured fidelity to the intervention itself but not to the implementation strategy, i.e., whether the strategy was executed as planned which could be considered in future evaluations [77–79]. It would also be interesting to observe how pathway fidelity is preserved or adapted over time, and what modifications to the implementation strategy are required to support long-term integration e.g., leadership and organisational processes.

Levels of adherence to clinical actions pre and post implementation highlight an important consideration when measuring or interpreting fidelity results in studies, particularly those with smaller samples. In the surgical cohorts that demonstrated no significant difference in compliance scores, the baseline rates of compliance were high for most variables being 80% or over. This ceiling effect likely limited the potential for measurable change compared to both orthopaedic cohorts, which had lower baseline compliance levels for more clinical actions (only 8 of 15 being 80% or over at baseline). These findings suggest that when adherence is already high, obtaining additional gains in fidelity may require targeting low-performing components specifically.

There were limitations to the study. This study was conducted in a private, metropolitan hospital and other settings may yield additional insights. While we strengthened validity through data triangulation, the number of clinicians interviewed was small, no surgeons consented to be interviewed, and self-selected participation may not be generalisable to the broader facility’s workforce. While BMI was identified as a potential confounder for the THA cohort, BMI data was not captured for spinal and radical prostatectomy cohorts and therefore not assessed. The timing of post-implementation data collection at 12 months post implementation may not have captured longer-term sustainment effects or the gradual embedding of practices over time. Finally, the organisation identified variation as an issue within the orthopaedic cohorts prior to commencing this formal project and had conducted a prior quality improvement initiative which informed aspects of this study [25]. Secular improvements in the orthopaedic cohorts may therefore have been occurring over time.

## Conclusions

This study demonstrates a practical example of how real-world, focused implementation efforts using implementation science tools can generate theory-informed insights through process evaluations. Utilising a flexible, multi-faceted implementation plan with local consensus discussions as the primary strategy, supported the integration of perioperative pathways into routine care in part. The EPIS framework was useful in identifying shifting contextual needs across implementation phases, while NPT provided a lens to understand how the intervention became embedded into practice and where mechanisms were lacking. Local consensus discussions were considered a useful and acceptable implementation strategy, particularly when adapted to the needs of each surgical team, helping involved clinicians to engage meaningfully in intervention delivery.

## Supplementary Information

Additional file 1. Standards for Reporting Implementation Studies (StaRI) checklist, (.pdf). Populated checklist of ‘Standards for Reporting Implementation Studies’ reporting guidelines indicating how the manuscript adheres to the relevant guidelines.

Additional file 2. Topic guide for semi structured interviews utilised for healthcare professionals, (.pdf). Provides the topic guide initially piloted by the research team and used to guide the interviewer in the semi-structured interviews for clinicians.

Additional file 3. Detailed quantitative analysis plan, (.pdf). Provides additional details and more detailed information pertaining to the quantitative analysis component of the study. 

Additional file 4. Table S1. Listing of clinical pathway actions included in medical record audits, (.pdf).

Additional file 5. Table S2. Implementation outcomes, themes and supporting quotations according to EPIS constructs, (.pdf).

## Data Availability

The datasets used and/or analysed during the current study are available from the corresponding author on reasonable request.

## Abbreviations

BMI: Body Mass Index
EPIS: Exploration Preparation Implementation Sustainment Framework
ERAS: Enhanced Recovery After Surgery
IQR: Interquartile range
NPT: Normalisation Process Theory
RAPT: Rapid Assessment and Prediction Tool
SD: Standard deviation
THA: Total Hip Arthroplasty
TKA: Total Knee Arthroplasty
