# Supplementary material for: Conducting rigorous implementation evaluations in real word settings: lessons from a consensus approach to perioperative pathway implementation for elective surgery
Source: Implement Sci Commun. 2026 Feb 6;7:46. doi: 10.1186/s43058-026-00876-4 (PMC12977520; doi:10.1186/s43058-026-00876-4)
Supplement: Supplementary file 2 — Additional file 2. Topic guide for semi structured interviews utilised for healthcare professionals, (.pdf). Provides the topic guide initially piloted by the research team and used to guide the interviewer in the semi-structured interviews for clinicians. [file 43058_2026_876_MOESM2_ESM.docx]

**Topic Guide: Surgeons**

| **Title** | Implementation of evidence and consensus-based perioperative care pathways |
| --- | --- |

The semi-structured interviews will be conducted retrospectively for key clinical and non-clinical hospital staff who were previously involved in the development of perioperative pathways for the elective hip and knee replacement surgical cohorts; and prospectively for staff currently involved in the development and implementation of new pathways. The aims of the interview are to:

- Determine the experiences of staff involved in implementing standardised peri-operative pathways.
- Explore the stakeholder knowledge of the care pathways within the hospital setting.
- Assess the integrity, fidelity to and feasibility of the intervention.
- To understand the key barriers and facilitators to implementation of standardised peri-operative pathways from the perspective of both clinical and non-clinical hospital staff.
- To synthesize data from both patient groups to gain a broader understanding of how standardised care pathways are implemented and the impact of these pathways.

**QUESTIONS:**

1. Can you please introduce yourself and explain your role?
2. How long have you worked in healthcare?
3. How many of those have been at [Name of] Hospital?
4. What was your experience/what has been your experience implementing the [SURGICAL COHORT] peri-operative pathway/s?

Alternate - How do you understand that process was undertaken at the time?

1. How familiar are you with the particulars of the clinical pathway/s? Can you talk through some of the changes from previous practice?

- What worked?
- What didn’t work?

1. What are your views on the standardised clinical pathways that were implemented/are being implemented?

- Strengths
- Weaknesses

1. What are your perceptions of why [organisation] was looking to standardise pathways?
2. What challenges, if any, did you encounter in implementing the clinical pathways?

Prompts;

- Workload and time
- Staffing
- Personal preference/views/autonomy
- Inter-professional collaboration
- Other

1. What was important to facilitating the implementation of the clinical pathways?

Prompts;

- Organisation
- Resources
- Staffing/Inter-professional collaboration
- Support/Monitoring of fidelity/quality
- Other

1. Where practitioners do differ in their clinical practice (for example DVT prophylaxis, Abx use: a) do you think it’s an issue? b) how might that be resolved?
2. What are your suggestions to improve future implementation of standardised clinical pathways for [SURGICAL COHORT] surgery?
3. What do you see as the effects and value of implementing standardised clinical pathways for [SURGICAL COHORT] surgery?
4. How would you measure the success of a clinical pathway of the [SURGICAL] clinical cohort?

1. Overall, how feasible is/was it to implement standardised clinical pathways for [SURGICAL COHORT] surgery [Organisation] or private hospital settings more generally?

1. Is there anything else you would like to add that has not been covered here?

**Topic Guide: Nursing/Allied health interviews**

| **Title** | Implementation of evidence and consensus-based perioperative care pathways |
| --- | --- |

The semi-structured interviews will be conducted retrospectively for key clinical and non-clinical hospital staff who were previously involved in the development of perioperative pathways for the elective hip and knee replacement surgical cohorts; and prospectively for staff currently involved in the development and implementation of new pathways. The aims of the interview are to:

- Determine the experiences of staff involved in implementing standardised peri-operative pathways.
- Explore the stakeholder knowledge of the care pathways within the hospital setting.
- Assess the integrity, fidelity to and feasibility of the intervention.
- To understand the key barriers and facilitators to implementation of standardised peri-operative pathways from the perspective of both clinical and non-clinical hospital staff.
- To synthesize data from both patient groups to gain a broader understanding of how standardised care pathways are implemented and the impact of these pathways.

**QUESTIONS:**

- Can you please introduce yourself and explain your role?
- How long have you worked in healthcare?
- How many of those have been at [Name of] Hospital?
- What was your experience/what has been your experience implementing the [SURGICAL COHORT] peri-operative pathway/s?
- How familiar are you with the particulars of the clinical pathway/s? Can you talk through some of the changes from previous practice?
- What worked?
- What didn’t work?
- What are your views on the standardised clinical pathways that were implemented/are being implemented?
- Strengths
- Weaknesses
- Did you and other clinicians tend to follow the new processes/procedures?
- Why do think that is?
- What challenges, if any, did you encounter in implementing the clinical pathways?

Prompts;

- Workload and time
- Staffing
- Personal preference/views/autonomy
- Inter-professional collaboration
- Other
- What was important to facilitating the implementation of the clinical pathways?

Prompts;

- Organisation
- Resources
- Staffing/Inter-professional collaboration
- Support/Monitoring of fidelity/quality
- Other
- Were there any other lessons learned about implementing standardised clinical pathways?

*Additional probing question if not covered in answer above:*

- What are your suggestions to improve future implementation of standardised clinical pathways for [SURGICAL COHORT] surgery?
- What do you see as the effects and value of implementing standardised clinical pathways for [SURGICAL COHORT] surgery?
- Overall, how feasible is/was it to implement standardised clinical pathways for [SURGICAL COHORT] surgery at [Organisation] or private hospital settings more generally?
- Is there anything else you would like to add that has not been covered here?

**Topic Guide: Management**

| **Title** | Implementation of evidence and consensus-based perioperative care pathways |
| --- | --- |

The semi-structured interviews will be conducted retrospectively for key clinical and non-clinical hospital staff who were previously involved in the development of perioperative pathways for the elective hip and knee replacement surgical cohorts; and prospectively for staff currently involved in the development and implementation of new pathways. The aims of the interview are to:

- Determine the experiences of staff involved in implementing standardised peri-operative pathways.
- Explore the stakeholder knowledge of the care pathways within the hospital setting.
- Assess the integrity, fidelity to and feasibility of the intervention.
- To understand the key barriers and facilitators to implementation of standardised peri-operative pathways from the perspective of both clinical and non-clinical hospital staff.
- To synthesize data from both patient groups to gain a broader understanding of how standardised care pathways are implemented and the impact of these pathways.

- Can you please introduce yourself and explain your role?
- How long have you worked in healthcare?
- How many of those have been at [Name of] Hospital?

- How did you get involved in this project?
- What is your primary role in this project?
- What are your perceptions of why [Organisation] was looking to standardise pathways?
- What was your experience/what has been your experience developing/implementing the [SURGICAL COHORT] peri-operative pathway/s?
- How familiar are you with the particulars of the clinical pathway/s? Can you talk through some of the changes from previous practice?
- What worked?
- What didn’t work?
- What are your views on the standardised clinical pathways that were implemented/are being implemented?
- Strengths
- Weaknesses
- What challenges, if any, did you encounter in implementing the clinical pathways?

Prompts;

- Workload and time
- Staffing
- Personal preference/views/autonomy
- Inter-professional collaboration
- Other

- What was important to facilitating the implementation of the clinical pathways?

Prompts;

- Organisation
- Resources
- Staffing/Inter-professional collaboration
- Support/Monitoring of fidelity/quality
- Other
- What are your suggestions to improve future implementation of standardised clinical pathways for [SURGICAL COHORT] surgery?
- What do you see as the effects and value of implementing standardised clinical pathways for [SURGICAL COHORT] surgery?
- How would you measure the success of a clinical pathway of the [SURGICAL] clinical cohort?

- Overall, how feasible is/was it to implement standardised clinical pathways for [SURGICAL COHORT] surgery at [Name of] Hospital or private hospital settings more generally?

- Is there anything else you would like to add that has not been covered here?
