# Supplementary material for: Conducting rigorous implementation evaluations in real word settings: lessons from a consensus approach to perioperative pathway implementation for elective surgery
Source: Implement Sci Commun. 2026 Feb 6;7:46. doi: 10.1186/s43058-026-00876-4 (PMC12977520; doi:10.1186/s43058-026-00876-4)
Supplement: Supplementary file 3 — Additional file 3. Detailed quantitative analysis plan, (.pdf). Provides additional details and more detailed information pertaining to the quantitative analysis component of the study. [file 43058_2026_876_MOESM3_ESM.docx]

**Additional File 3: Detailed Quantitative Analysis Plan**

Quantitative fidelity data were extracted and imported into the Statistics Package for Social Sciences (SPSS) Version 29.0 for analysis. For each pathway element, pathway adherence (dosage) was assessed as the percentage of patients that were compliant (1). Each audited clinical action was first scored as 0 for non-compliance or 1 for compliance and then summed for each patient in each audit year (2022, 2023, and 2024) to give a patient compliance score. For example, for total knee arthroplasty (TKA) there were 15 pathway components, so the score for any patient could range from 0 (no compliant components) to 15 (all components compliant). Total hip arthroplasty (THA) also had a maximum score of 15, while radical prostatectomy had a maximum score of 11 and spinal surgery a maximum score of seven. These measures can be used to determine intervention fidelity (dosage) and to provide an overall measure of pathway compliance.

Descriptive statistics were calculated to describe the distribution of individual patient pathway compliance scores (mean [SD], median [IQR], minimum, maximum) by cohort (n=4) and year (n=3). The distribution of scores for the target outcome was assessed for normality by visual inspection of a histogram, separately for the pre- and post-intervention periods; given the small sample size. By visual inspection, none of the pathway compliance score distributions were deemed normal, and this was confirmed by statistical tests of non-normality which were all statistically significant regardless of the small sample sizes.

The primary analysis was specified as a two-sample t-test, comparing the pre- and post-intervention pathway scores, as the t-test is robust to deviations from normality (2). As the official implementation date for the intervention was the 1^st^ of November 2023, and some changes were likely to have occurred earlier in anticipation, all of 2023 was considered a transition year. The primary analysis compared 2022 (pre-intervention) with 2024 (post-intervention). Transition year data was perused to give an indication of the extent to which pathway component compliance had been anticipated by practice, to help understand how change happens in the real world.

To identify which clinical actions contributed to observed changes, a planned post-hoc analysis was conducted on cohorts where the primary analysis showed a statistically significant difference between the means. The proportion of patients who received each clinical action was calculated to permit detailed analysis of which components changed over time for this post-hoc analysis. Fishers exact test was used to assess statistical significance, with the significance level set at p<0.05.

Secondary analysis was conducted to assess for the potential impacts of confounding of baseline clinical risk. Due to the small sample size, we created simple rules of thumb to identify potential confounders. There were one to three variables assessed in each cohort: age (all cohorts); sex (all cohorts except prostate surgery); and Body Mass Index (BMI) (for THA and TKA only). Due to a lack of literature to determine whether a demographic variable was a known confounder and to prevent overfitting of models for multivariable analyses, potential confounding was purposefully screened and selected by assessing their maldistribution between the pre- and post-intervention periods; or assessing their causal link as a risk factor for pathway adherence score. For assessing maldistribution, pre- and post-intervention periods were compared for each cohort. For the binary variable sex, a difference of 10 percentage points (absolute change) was considered sufficient to require assessment of confounding (e.g., assess for 60% vs 80%, but not 60% vs 69%). For continuous variables (age or BMI), a 10% relative difference in median was sufficient to require further consideration. For assessing risk-factor status we collapsed all three cohorts (2022-2024) to increase the sample size. As the distribution of total pathway adherence scores in all cohorts was non-normal, univariable quantile regression of the median was conducted to examine if the variables were potentially predictive of outcome and test whether there was any link between potential confounders and pathway scores. A threshold of p<0.2 was used to indicate potential risk-factor status, similar to the thresholds advocated for consideration of variables when building predictive models (3). Continuous explanatory variables (age and BMI) were first broken into quartiles and the average pathway score for each quartile was calculated to determine the best shape to include in the model (e.g., as a linear, quadratic, or categorical explanatory variable).

For variables that were found to be potential confounders since they were either predictive of outcome according to our screening threshold or were maldistributed, assessment of confounding was then conducted. The univariable model comparing 2022 and 2024 results was used to generate the estimated impact of the perioperative pathways. A bivariable model was then fit adding each of the screened confounders and an adjusted estimate calculated. If the estimate of effect (post- vs pre-intervention) changed by 10% (relative change) (4) and a minimum of 0.5 points (absolute score change),the potential confounder was retained. Where two or more proven confounders were found in bivariable analysis, each additional variable needed to result in a 10% relative change (and 0.5-point absolute change) for that variable to be retained in the final multivariable models. The point estimate for impact of perioperative pathways after adjustment for all demonstrated confounders were reported.

**Abbreviations:**

BMI: Body mass index

THA: Total hip arthroplasty

TKA: Total knee arthroplasty

**References:**

1. Carroll C, Patterson M, Wood S, Booth A, Rick J, Balain S. A conceptual framework for implementation fidelity. Implement Sci. 2007 Nov 30;2:40. doi: 10.1186/1748-5908-2-40
2. Havlicek LL, Peterson NL. Robustness of the t test: A guide for researchers on effect of violations of assumptions. Psychological Reports. 1974 Jun;34(3_suppl):1095-114.
3. Hosmer Jr, David W., Stanley Lemeshow, and Rodney X. Sturdivant. *Applied logistic regression*. John Wiley & Sons, 2013.
4. Budtz–Jørgensen, E., Keiding, N., Grandjean, P., & Weihe, P. (2007). Confounder selection in environmental epidemiology: assessment of health effects of prenatal mercury exposure. *Annals of epidemiology*, *17*(1), 27-35.
