# Supplementary material for: Conducting rigorous implementation evaluations in real word settings: lessons from a consensus approach to perioperative pathway implementation for elective surgery
Source: Implement Sci Commun. 2026 Feb 6;7:46. doi: 10.1186/s43058-026-00876-4 (PMC12977520; doi:10.1186/s43058-026-00876-4)
Supplement: Supplementary file 4 — Additional file 4. Table S1. Listing of clinical pathway actions included in medical record audits, (.pdf). [file 43058_2026_876_MOESM4_ESM.docx]

**Table S1: Listing of clinical pathway actions included in medical record audits**

| **Pathway variable** | **Cohort** | | | |
| --- | --- | --- | --- | --- |
|  | **THA** | **TKA** | **Radical prostatectomy** | **Spinal surgery** |
| BEFORE SURGICAL ADMISSION |  |  |  |  |
| Attended PAC | ☑ | ☑ |  |  |
| RAPT form completed | ☑ | ☑ |  |  |
| PAC attendance within 3-4 weeks of day of surgery | ☑ | ☑ |  |  |
| Physiotherapy review at PAC appointment | ☑ | ☑ |  |  |
| Patient health questionnaire completed | ☑ | ☑ | ☑ | ☑ |
| DURING SURGICAL ADMISSION |  |  |  |  |
| Admissions tab completed | ☑ | ☑ | ☑ | ☑ |
| Admitted on day of surgery | ☑ | ☑ | ☑ | ☑ |
| VTE assessment completed on day of surgery | ☑ | ☑ | ☑ | ☑ |
| VTE compliance^1^ | ☑ | ☑ | ☑ | ☑ |
| Planned discharge destination identified preoperatively | ☑ | ☑ |  |  |
| SAP according to guidelines | ☑ | ☑ |  |  |
| Mobility compliance^2^ | ☑ | ☑ | ☑ | ☑ |
| IDC compliance^3^ | ☑ | ☑ | ☑ |  |
| Patient controlled analgesia compliance^4^ | ☑ | ☑ |  |  |
| CNC review during admission |  |  | ☑ |  |
| Discharge summary received by date of discharge | ☑ | ☑ | ☑ | ☑ |
| AFTER SURGICAL ADMISSION |  |  |  |  |
| CNC review within 2 weeks of discharge |  |  | ☑ |  |
| Prostate cancer nurse review within 1 month of discharge |  |  | ☑ |  |

Abbreviations: CNC=Clinical nurse consultant; IDC=indwelling catheter; PAC=preadmission clinic; Preop=preoperatively; POD = postoperative day; RAPT= Risk assessment and Prediction Tool; SAP=surgical antibiotic prophylaxis; THA=Total hip arthroplasty; TKA=Total knee arthroplasty; VTE=venous thromboembolism

Notes: 1 Considered compliant if patient was prescribed both Thrombo-Embolic Deterrent stockings (TEDs) and Sequential Compression Devices (SCDS); 2 For total hip arthroplasty/total knee arthroplasty, considered compliant if patient was mobilised on Post-operative Day 0. For spinal surgery/radical prostatectomy, considered compliant if patient mobilised on Post-operative Day 0 or 1; 3 For total hip arthroplasty/total knee arthroplasty, considered compliant if patient did not have an IDC or if IDC was removed within 24 hours of surgery. For radical prostatectomy, considered compliant if IDC was changed to a leg bag prior to day of hospital discharge; 4 Considered compliant if patient did not have a patient-controlled analgesia or if patient-controlled analgesia was removed within 48 hours of surgery
