# Supplementary material for: Conducting rigorous implementation evaluations in real word settings: lessons from a consensus approach to perioperative pathway implementation for elective surgery
Source: Implement Sci Commun. 2026 Feb 6;7:46. doi: 10.1186/s43058-026-00876-4 (PMC12977520; doi:10.1186/s43058-026-00876-4)
Supplement: Supplementary file 5 — Additional file 5. Table S2. Implementation outcomes, themes and supporting quotations according to EPIS constructs, (.pdf). [file 43058_2026_876_MOESM5_ESM.docx]

**Table S2. Implementation outcomes and themes according to EPIS constructs**

| **Implementation outcome^1^** | **EPIS construct^2^** | **Descriptive inductive theme** | **Example from observation or interview** |
| --- | --- | --- | --- |
| **Acceptability**  *The perception among implementation stakeholders that a given treatment, service, practice, or innovation is agreeable, palatable, or satisfactory* | Innovation factors, Innovation/EBP characteristics | Facilitates adherence to evidence-based practice  Establishes benchmarks for clinical care  Promotes positive outcomes for patients | “It actually facilitated some things to happen like patients were, getting up day zero or the patients needed to have their IDC out day one, you know barriers that I found as a physiotherapist […] rather than a nurse saying to me, ‘the patient doesn’t want to get it out’ I was able to then ‘this is the pathway’, that's what we've agreed upon doing […] this is what we should be following it's best practise it’s evidence based.” [Int 4, clinician]  “I think that we would stand to benefit from that point of view if we were providing this minimum kind of care, as a benchmark, that this is where all our surgeons are going to perform, that this is what you could expect as an outcome for this procedure coming to us” [Int 8, management]  “I found was that the patients got up earlier, they looked better and they had better analgesia. So from you know that which is my main aim in for my anaesthetic practise […] they should look good. No nausea and vomiting, they should be pain free […] So when surgeons started seeing that overall by using different you know by standardised techniques, yes, then they started buying in more and more […] the divergences reduce and they started converging into the standard pathways because they put pressure on their anaesthetists to actually change their practice too.” [Int 5, clinician] |
|  | Innovation factors,  Innovation/EBP developers | Broad medical representation  Multidisciplinary representation and involvement  Promotes acceptability  Limited multidisciplinary involvement in implementation  Lack of engagement with frontline staff leading to a disconnect from actual pathways  Early inclusion of all disciplines to ensure fidelity and address flow-on effects | “If I if we had a pathway that was written entirely by someone like [surgeon] and [anaesthetist]. Yeah, well, you only have one person. You're gonna get 100% agreement, but by the time you go to the broader discipline and say this is what we've come up with, people are gonna say well, you know like it's one person’s world view, so I'm just going to continue to stick to my practise. So you kind of need to have um, your your [surgeon X] there, but you also need to have your [surgeon Y] there, or someone who's very respected in particular around the infection control prevention control space. Yeah, you potentially need someone like a [surgeon z] there who is very evidence focused and knows the most recently published papers so if you can come up with a document that say, these four disparate people, each with their own strengths, brings to the table, you're far more likely to get something that everyone says OK, I'm more likely to kind of follow that” [Int 1 management]  “So for me where I sit in the organisation, I feel there's been appropriate consultation […] However, perhaps not enough at that […] patient facing level [..]. I think getting feedback from the ground level would be really beneficial because I think that's where we're going to see a lot of impact of the implementation and how we can improve or change what we're doing to get those outcomes.” [Int 8 management] |
|  | Innovation factors,  Innovation/EBP fit: system, organisation, provider, patient | Opportunity to improve hospital processes  Streamline care  Obtain useful data  Empowers staff  Opportunity to improve patient education processes | “Let's see if we can bring it more streamlined so that the instructions are the same. The team understand the same, so they're not trying to have preference cards for 15 people for 15 procedures” [Int 3, management]  The facilitator seems to be successful in getting the room on board with the usefulness and importance of the pathways through the data that can be produced and repeating that there is an opportunity to have a consistent standard of care. [Nurse X] says enthusiastically “I think it’s really helpful, I have 15 different versions of this [pointing to the pathway] and that’s unhelpful to me because […] patients always say ‘well my doctor says’ and ‘I was booked for 5 days so I’m staying those 5 days’”. [Observation, Spinal, Oct 2023]  “Where you have to be the one to provide the care, being conscious of those preferences can delay your actions and decision making […] and you're less empowered to make decisions because ‘ohh, hang on, I'm not really sure what that actual specialists preference is, I need to go and check’ and then you go and check, well, that's actually not how I would think this is the best outcome or the best for the patient and then there's that conflict. So I feel like this is a really good process.” [Int 8 management]  “I don't think it was even like it was recorded in our notes, but there was no collection of information to look at why is length of stay so long? […] Is the delay because everyone's going to rehab and rehab has no beds and everyone's being backlogged from going? Is it because of medical? […] I felt there was also opportunity to collect information that can help improve care.” [Int 4, clinician]  The facilitator says another positive outcome of developing the pathways is how it provides the hospital with the opportunity to revise patient education materials observation [Observation, Nurse Leaders Committee Meeting, Nov 2024] |
|  | Inner context,  Individual characteristics | Satisfaction with the intervention and with consultation  Resistance to change and barriers intervention acceptance  Opposing opinions amongst surgeons  Desire for autonomous practice | “I think there was good buy in. Yeah, I think they were quite like enthusiastic about it.” [Int 8, clinician]  “Cultural change was also difficult. OK, so if you're changing, if you'll be doing one particular practise for years on end, you know, it's hard for them to change their practise and actually see the relevance of that, because sometimes you can't see the relevance of your change in practise initially […] And so that that was quite frustrating and some people just don't want to change.” [Int 5, clinician]  Considering that the dressings have been standardised in this document, the question is asked if the discipline have seen this, and the facilitator says that the discipline lead has signed off on it and it has been circulated around to the others so they have all seen it. The facilitator then says “I don’t know what the response will be” to the change in practice. [Observation, Spinal, Nov 2023]  Referring to surgeons “You know with wound dressings, “Oh no, this is the practise that we know, so we're not going to change it” like you know, you just sent your nurses to a study day about that and then you get a surgeon who, has their own practise […] which is really hard to navigate. So yeah, something like a pathway that will be approved by the department is a good way going forward.” [Int 7, clinician/management] |
|  | Inner context,  Quality & fidelity monitoring/support | Clinician engagement in outcome evaluation  Prioritising and capturing clinician-driven outcome measures | [Facilitator] then asks the “subject matter experts” what are some other key outcomes that the discipline would want audited so that the organisation can set that up without having to trawl through every single Electronic Medical Records note for every detail. Surgeon Z says that drain education given at preadmission visit and postoperative days 1 and, as well as phone calls about drains. [observation, breast surgery, Oct 2024]  “Nurse Y says that length of stay is not a useful outcome for them but highlights how staff shortages and patient flow is important. At this point, Nurse Z says that resources are important to measure or address. [Observation, cardiac, Mar 2024] |
| **Adoption**  *The intention, initial decision, or action to try or employ an innovation or evidence-based practice* | Outer context,  Inter-organisational environment & networks | Commitment, competition and desire to make change.  Alignment with peer organisations as a catalyst for change | The facilitator continues on about how “our performance may not have been the same” as peer organisations and so it was in the interest of the organisation to start this work. [Observation, breast surgery, Mar 2024]  “I think that we would stand to benefit from that point of view if we were providing this minimum kind of care, as a benchmark, that this is where all our surgeons are going to perform, that this is what you could expect as an outcome for this procedure coming to us.” [Int 8, clinician] |
|  | Inner context,  Leadership | Need for discipline leadership and support  Supporting clinicians to change behaviour  Formal versus informal influence  Importance of implementation leadership  Implementation Champions drive change through presence and familiarity | Each of the successful pathways has had a leader and discipline leadership. [Observation, cardiac, Mar 2024]  The facilitator says that when they attempted to create the pathways last time (ie 2 years ago), whilst they did a significant amount of groundwork, they encountered challenges with leadership from both the nursing side and also at the medical level so “it sort of went nowhere and attentions got allocated elsewhere.” […] He raises that there is a need for leadership engagement with the process and the establishment of a consensus group. [observation, cardiac, Mar 2024]  The facilitator repeats an interesting anecdote from the anaesthetics team about how one of the main drivers for increased length of stay for the orthopaedic cohort was that people were getting prophylactically put on the intensive care unit list before being admitted for surgery due to having small risk factors. When the head of discipline heard this, he made a directive to the rest of the group to basically stop doing this and length of stay reduced significantly for this patient cohort following that directive. [Observation, nurse committee meeting. Nov 2023]  “And to a certain extent, you, you can drive the priorities of the people who you employ. So it's it's easy enough for me to say to, for example, to the senior physiotherapist in Orthopaedics “this is important work and you need to be dedicating time and energy and effort to try to make this, um, work.” But for people that you don't have an employment relationship with, it, you are to some extent relying on good will, and and a, a ahh, sense of responsibility to a broader focus rather than just an individual's, clinical practise.” [Int 1, management]  “But it's funny that I don't hear anything more about: how we're gonna do this or, you know, what is the plan. Is there going to be education, how how are we going to, you know, meet this expectation supporting these pathways in our departments. There was a kind of a little bit of that in that meeting, but there's been nothing, no follow up.” [Int 8, clinical management] |
|  | Inner context,  Individual characteristics | Enthusiasm and engagement with the process  Surgeon engagement and incentives to promote engagement  Surgeons influence peers to adopt and spread practice changes.  Outcomes can act as an incentive to promote adoption | “And of course, you know people who have got responsibilities to other organisations and responsibilities to clinical practise and that whole kind of […] what Macquarie University Hospital is doing as an institution. Some people will prioritise that and be able to dedicate time to that and other people will be dedicating that time and priority to other organisations where they work.” [Int 1, management]  “It basically comes down to. Yeah, the surgeon. OK and the surgeon and how they really wanted to see their practise because they you know. And so, yes, there were people who you know, because this information is all being passed on through a lot of the orthopaedic surgeon community. So if they wanted that style of practise, you know, they push their anaesthetists, they push their staff, they push their protocols towards that end, and those people who wanted individual practise, well, they just did what they wanted to do.” [Int 5, clinician]  “How do we influence people to, perhaps practise in a way that's more according to the pathway? Well, perhaps let's present data to the discipline more broadly, which is the identifier that kind of says, well, you know, this is what the discipline did as a whole. And then maybe I'll go back to individual practitioners, be they surgeons, be they anaesthetist or or whoever. And kind of say, well look, this is, where your patients have gone, this is where the cohort sits overall.” [Int 9, management] |
|  | Innovation factors,  Innovation/EBP developers | Individual drive to be involved affects engagement and motivation | “OK, so we're trying to engage now, senior nursing, senior allied health in the development of both so that they understand the kind of the development where it lands. So that we hope there will be, you know, agreement to kind of implement that pathway on the ward.” [Int 1, management] |
| **Appropriateness**  *Perceived fit, relevance, or compatibility of the innovation and/or perceived fit of the innovation to address a particular problem* | Outer context,  Patient/client characteristics | Differing expectations of care or outcomes | Nurse X says enthusiastically “I think it’s really helpful, I have 15 different versions of this [indicating to the pathway] and that’s unhelpful to me because […] patients always say “well my doctor says” and “I was booked for 5 days so I’m staying those 5 days” so I think this is really helpful.” Nurse Y reiterates the same positivity commenting that “often people ask ‘Do I have to go home?’ and the doctor says ‘well you don’t have to’ so that creates a lot of problems [for the staff]” to which Nurse Z agrees “yeah it’s so annoying.” [observation, Spinal implementation, Oct 2023] |
|  | Innovation factors,  Innovation/EBP characteristics | Appropriate to address unwarranted clinical variation  Appropriate to address issues related to variation and promote adherence to EBP e.g. clinician preference or concerns | “If you've got 15 different preferences, then that can lead to confusion and lead to mixed messaging and people, you know, getting the incorrect thing. Whereas if you have one standard approach, very easy, everyone's on the same page you know how to do it.” [Int 1, management]  “But sometimes I think being a clinician on the ground, it can be a bit challenging, if you've got different protocols from different things and you're like, oh, wait, this person will do this first and then day two, I have to do this whereas this person is bed rest and it can be quite challenging, yeah. So, you can imagine how more efficient that becomes because everybody's informed everybody knows what's happening” [Int 3, management]  “Whereas I think people that were maybe in my position, looked at it as no, it's a pathway, so it actually facilitated some things to happen like patients were, you know, getting up day zero or the patients needed to have their IDC out day one, you know barriers that I found as a physiotherapist, I had a little bit more justification and say you know, rather than a nurse saying to me, oh, you know, all the patient doesn’t want to get it out. I was able to then say look this is the pathway, that's what we've agreed upon doing.” [Int 4, clinician] |
|  | Inner context,  Organisational characteristics | Structures within private hospitals | “So private hospital might be a little bit different to some of the public hospital settings that you don't necessarily […] have that capacity for regular interaction between all of the practitioners in a particular area. And once you get into the private sector, most of the people practising are specialists […] there's probably an assumption that, that they are doing things the right way […] that they are probably practising precisely the same way as their peers. I don't think there's necessarily the regular opportunities for these things to be canvassed more broadly and to ask the question you know - what do you do for VTE prophylaxis? […] When people do get together, it's often for mortality and morbidity meetings […], rather than the routine elements of good practise standardised practise” [Int 9, management] |
| **Feasibility**  *The extent to which a new innovation can be successfully used or carried out within a given setting* | Innovation factors,  Innovation/EBP fit: system, organisation, provider, patient | Ability of the implementation process to be adapted to fit discipline needs | The facilitator acknowledges that staff may have ideas for aspects of the pathway that they may want to change to suit implementation. He appears to acknowledge that different methods of implementation will work for different staff disciplines and says that the “options are open for what we choose to do for implementation” opening the floor up to the room. [Observation, spinal implementation, Oct 2023] |
|  | Innovation factors,  Innovation/EBP developers | Inclusion of multidisciplinary perspectives enables consideration of workflows, streamlines implementation and enhances buy-in  Successful pathway development requires multidisciplinary collaboration and coordinated engagement | “The problem there is not necessarily an issue of will from the nursing staff to adhere to the pathway […] it's more a case of, well, we might want to do this, but we're hamstrung to a certain extent because our patients are having anaesthetic approaches, which means that if we take the catheters out too early, inevitably we find we're gonna have to put them back in … And so it it's challenging for us to fit that in with our workflow when we've got so many patients to look after.” [Int 1, management]  In answer to who needs to be in the room, Nurse Y comments that with the joint surgeries “anaesthetists have a huge impact on it, they’re the ones that change the pathway a lot.” [Observation, orthopaedic, Nov 2023] |
|  | Inner context,  Leadership | Importance of implementation leadership  Implementation champion has knowledge of the organisation  Implementation champions from different clinical levels of care | Referring to previous projects “So what was helpful, I think is having someone in the project and steering that, but at the same time that person isn't a clinician working there, so […] I felt it was also hard because they truly didn't know actually how things ran on the ward […] To fix it, pay for a clinician to do it […] [referring to this project] So for example [the facilitator] yeah, being involved, he's been here for a long time. He has worked on the wards you guys have. He has a good understanding and so therefore I think that really is better because he understands probably the nuances of the differences and understands the demands on the ward.” [Int 7, clinician]  “Once the pathway has been completed […] we need to talk to the consulting rooms. We need to talk to the Physio Department. We need talk to peri-assessment department. We need to talk about to theatre, procedure, your post op in the ward. So there needs to be a champion in each of those areas that's, that working party before you can ever implement it. You have to have it accountable.” [Int 3, management] |
|  | Inner context,  Individual characteristics | Clinicians are time-poor  Low individual knowledge of pathways | Logistics are mentioned and the difficulties in having surgeons collect this data. Surgeon C explains “The hardest part is getting surgeons to do their part […] so you need to make it as easy as possible.” [observation, spinal, Mar 2023]  Team education is needed and the need to get everyone on the same page. A hypothetical anecdote is used as an example by Nurse X where a staff member goes into a patient’s room and says “Oh so you had your surgery on Friday so you’ll probably go home next Friday or are you going to rehab?” which can be a challenge. [Observation, Spinal, Nov 2023] |
|  | Inner context,  Organisational characteristics | Logistical factors impacting implementation  Who should be responsible to manage specific pathway components?  Administrative processes for different patient groups and surgical disciplines  Resource coordination  Inadequate technological resources to support implementation | The facilitator asks “Does there need to be an item in the pathway about checking booking forms?” They all agree emphatically however, when asked who should be responsible for this role as administrative staff would not be able to complete that task, they are quiet. Nurse S replies that “it’s the nursing unit managers who do it but that she’s not sure if that’s the ‘best place’ to do it.” [Observation, cardiac, Apr 2024]  “So we really need to look at how we communicate to the patients from the doctor's rooms through to our pre assessment clinic through to admissions bookings and day of day of procedure, knowing that they're actually going to be mobilised day of surgery. A lot of people wouldn't even understand that” [Int 3, management]  The facilitator then highlights how the neurosurgery department within the hospital only captures 10/15 surgeons which presents a problem with coordination of resources as not all surgeons are affiliated so not all patients will go through the same avenue. [Observation, spinal, Oct 2023]  “[The facilitator] has mentioned about the track care not being able to, to do, have that capability or something which you think it would after a while. And I remember nurses have mentioned about like the extra workload of then having to scan and stuff so you do really want those technology resources to help it” [Int 8, management] |
|  | Inner context, Organisational staffing processes | Siloed practices and a lack of collaboration between nursing, allied health, and doctors  Challenges arising from staffing levels  Time constraints create competing demands | Comments about the medical registrars being particularly problematic with this are raised in comparison to junior medical officers who just do what they’re told. There is a strong need to get registrars on the same page as “they usually make the flippant comments.” [observation, spinal implementation, Nov 2023]  The facilitator says that “everyone just assumes that what they do is normal practice and everyone else is doing the same” when you go to those mortality and morbidity meetings. Nurse Y then says “they have no knowledge of what happens on the wards…they talk more about theatre stuff” as well as cases that go poorly but there is very little knowledge or engagement outside of that. [observation, spinal implementation, Nov 2023]  “When you're really short staffed then you know your priorities as a nurse for the days to look after the patient, making sure they stay safe so um, my general comments around that, yes, there could be some challenges when we are we are short, or we have issues with resources.” [Int 7, clinician]  The facilitator says that he’ll give everyone a few more minutes to arrive and Surgeon X responds “let’s just get started as we’re right in the middle of a clinic” to which the facilitator concedes and begins the meeting. [observation, orthopaedic, Mar 2023] |
|  | Inner context,  Quality & fidelity monitoring/support | Ongoing education, feedback and support to clarify expectations and build competence  Feedback promotes individual behaviour change | It is noted that training should include that nursing staff can mobilise patients. The facilitator raises the complications with cardiovascular instability and vasovagal events etc. and how the worst thing would be to have a patient fall and fracture a hip so strong education is needed around these points. [observation, orthopaedic, Nov 2023]    The facilitator mentions that one surgeon involved in the pathways development said to encourage behaviour change with surgeons, you need to show them where they sit individually on the scale as other surgeons are only interested in data if you show people their individual results rather than the discipline overall. [observation, spinal, Oct 2023] |
|  | Bridging factors, Community academic partnerships | Assistance from researchers | The facilitator mentions the usefulness of having researchers on board to help and providing a bit more manpower to start off with to help extract that data. [Observation, spinal, Mar 23] |
| **Sustainability**  *The extent to which a newly implemented treatment is maintained or institutionalized within a service setting’s ongoing, stable operations* | Inner context,  Quality & fidelity monitoring/support | Value of an ongoing audit and feedback process | “I think you can't implement something and then just assume it's there. But then also I think people find if you give them feedback and show them what the value of the tool is, people will be then reinforced […] so by providing that feedback from auditing and data collection, I think that improves the compliance with the tool.” [Int 4, clinician] |
|  | Innovation factors,  Innovation/EBP fit: system, organisation, provider, patient | Strategy adaptability to context for effective implementation? Can modify the approach to suit the context to optimise implementation | “We're kind of of letting them almost run the way that they're going to run rather than trying to impose or mandate a particular way that discipline might manage it. It’s kind of like, well, OK, if you think that'll work best for your discipline, let's try it in that way.” [Int 9, management] |
|  | Inner context,  Organisational characteristics | Balancing sustainability and workload in outcome collection  Challenges in monitoring implementation fidelity  Adequate resources needed | “And it's time consuming to collect. To be able to draw that data from it. So I'm all for data collection and review and auditing, provided that it's not just dumped on a clinician to expect them to do that.” [Int 4, clinician]  “Just don't want to see a situation where we've moved really nicely into a nice sort of streamlined pathway and then we start adding to it. By excessive amount of paperwork, you know, and I just hope that … we don't start to lose the sight of where we need to be going with this.” [Int 5, clinician] |

All quotations and observations are de-identified
Notes:
1Definitions of implementation outcomes are adapted from: Proctor E, Silmere H, Raghavan R, Hovmand P, Aarons G, Bunger A, Griffey R, Hensley M. Outcomes for implementation research: conceptual distinctions, measurement challenges, and research agenda. Adm Policy Ment Health. 2011 Mar;38(2):65-76. doi: 10.1007/s10488-010-0319-7.
2EPIS constructs as defined in: Moullin, J.C., Dickson, K.S., Stadnick, N.A. et al. Systematic review of the Exploration, Preparation, Implementation, Sustainment (EPIS) framework. Implementation Sci 14, 1 (2019). <https://doi.org/10.1186/s13012-018-0842-6>.
